# A new *Plasmodium vivax* reference sequence with improved assembly of the subtelomeres reveals an abundance of *pir *genes

**DOI:** 10.12688/wellcomeopenres.9876.1

**Published:** 2016-11-15

**Authors:** Sarah Auburn, Ulrike Böhme, Sascha Steinbiss, Hidayat Trimarsanto, Jessica Hostetler, Mandy Sanders, Qi Gao, Francois Nosten, Chris I. Newbold, Matthew Berriman, Ric N. Price, Thomas D. Otto

**Affiliations:** 1 Global and Tropical Health Division, Menzies School of Health Research and Charles Darwin University, Darwin, Australia; 2 Malaria Programme, Wellcome Trust Sanger Institute, Hinxton, UK; 3 Eijkman Institute for Molecular Biology, Jakarta, Indonesia; 4 Laboratory of Malaria and Vector Research, National Institute of Allergy and Infectious Diseases, National Institutes of Health, Rockville, USA; 5 Jiangsu Institute of Parasitic Diseases, Key Laboratory of Parasitic Disease Control and Prevention (Ministry of Health), Jiangsu Provincial Key Laboratory of Parasite Molecular Biology, Jiangsu, China; 6 Shoklo Malaria Research Unit, Mahidol-Oxford Tropical Medicine Research Unit, Faculty of Tropical Medicine, Mahidol University, Mae Sot, Thailand; 7 Centre for Tropical Medicine and Global Health, Nuffield Department of Clinical Medicine, University of Oxford, Oxford, UK; 8 Weatherall Institute of Molecular Medicine, University of Oxford, John Radcliffe Hospital, Oxford, UK

**Keywords:** Plasmodium, vivax, genome, reference, subtelomere, pir, vir

## Abstract

*Plasmodium vivax* is now the predominant cause of malaria in the Asia-Pacific, South America and Horn of Africa. Laboratory studies of this species are constrained by the inability to maintain the parasite in continuous *ex vivo* culture, but genomic approaches provide an alternative and complementary avenue to investigate the parasite’s biology and epidemiology. To date, molecular studies of *P. vivax* have relied on the Salvador-I reference genome sequence, derived from a monkey-adapted strain from South America. However, the Salvador-I reference remains highly fragmented with over 2500 unassembled scaffolds.  Using high-depth Illumina sequence data, we assembled and annotated a new reference sequence, PvP01, sourced directly from a patient from Papua Indonesia. Draft assemblies of isolates from China (PvC01) and Thailand (PvT01) were also prepared for comparative purposes. The quality of the PvP01 assembly is improved greatly over Salvador-I, with fragmentation reduced to 226 scaffolds. Detailed manual curation has ensured highly comprehensive annotation, with functions attributed to 58% core genes in PvP01 versus 38% in Salvador-I. The assemblies of PvP01, PvC01 and PvT01 are larger than that of Salvador-I (28-30 versus 27 Mb), owing to improved assembly of the subtelomeres.  An extensive repertoire of over 1200 *Plasmodium* interspersed repeat ( *pir*) genes were identified in PvP01 compared to 346 in Salvador-I, suggesting a vital role in parasite survival or development. The manually curated PvP01 reference and PvC01 and PvT01 draft assemblies are important new resources to study vivax malaria. PvP01 is maintained at GeneDB and ongoing curation will ensure continual improvements in assembly and annotation quality.

## Introduction

Infection with *Plasmodium vivax* is associated with significant direct and indirect morbidity that impacts on the poorest communities of malarious countries, with an estimated annual global cost of $1-2.7 billion ^1– 3^. Accumulating reports of drug-resistant infection and life-threatening disease underscore the urgency to reduce the burden of *P. vivax* and ensure its ultimate elimination ^4– 8^. Efforts to contain *P. vivax* are constrained by a limited understanding of the parasite’s basic biology, in part owing to the inability to maintain this species in continuous *ex vivo* culture. Genetic studies provide an alternative approach to gain novel insights into the parasite from which epidemiological tools and therapeutic approaches can be developed for clinical application ^9– 17^. The rapidly declining costs of massively parallel sequencing technologies have made it feasible to undertake whole genome sequencing of hundreds of *Plasmodium* isolates, with recent population genomic studies of *P. vivax* revealing novel antimalarial drug resistance and vaccine candidates amongst other biological features of the parasite ^16, 17^. However, in order to achieve a comprehensive understanding of the structure and composition of the *P. vivax* genome, and to improve read mapping efforts to characterise genetic polymorphisms, a high quality reference genome(s) representative of naturally occurring patient isolates is essential.

The sequences of 5 monkey-adapted strains including the Salvador-I reference ^14^ and drafts of Brazil-I, India-VII, North Korea and Mauritania-I ^13^ have provided important resources for the vivax research community to investigate the core genome of *P. vivax*. However, over 60% of the genes in the published Salvador-I reference ^14^ (prior to curation by the authors) had unknown function, limiting insight into underlying biological mechanisms. Furthermore, assembly of the subtelomeric regions is highly fragmented in these strains, with Salvador-I comprising >2500 scaffolds. A subsequent draft assembly of a Cambodian patient isolate (C127) revealed 792 genes not present in Salvador-I, including 366 new *pir* ( *Plasmodium* interspersed repeat) genes ^11^. The *pir* genes are a highly variable multigene family present in all *Plasmodium* genomes investigated to date ^18^. The function of *pir*-encoded proteins (PIRs) remains poorly understood, although recent studies suggest roles in mechanisms associated with virulence. *In vitro* studies of *P. vivax* have demonstrated PIR ** encoded protein mediated cytoadherence to endothelial cells ^19, 20^ and a *P. chabaudi* mouse malaria model demonstrated red blood cell-binding properties consistent with roles in invasion and/or rosette formation ^21^. A further *P. chabaudi* study demonstrated that changes in the expression of the *pir* gene repertoire following mosquito passage may attenuate virulence ^22^. The sequence diversity amongst the *pir* genes in *P. vivax* suggests that different subfamilies may have different functions ^14^. The published Salvador-I reference sequence revealed 346 *pir* genes, including 80 fragments and/or pseudogenes, 10 subfamilies and 84 unassigned genes ^14^. In the most recent computational classification, Lopez *et al.* re-classified the Salvador-I *pir* genes, excluding members of 3 major subfamilies (A, D and H) but including previously unassigned genes, and re-defining 39 genes as encoding PIRs rather than hypothetical proteins ^23^. However, given the limited number of PIRs in Salvador-I, further characterisation is required using a reference(s) with a more complete set of genes.

To address the need of the vivax research community for a *P. vivax* reference with more comprehensive assembly and annotation, we used Illumina genomic data to establish a reference from a Papua Indonesian patient isolate (PvP01). Since *P. vivax* exhibits marked regional variation in phenotypes such as duration of the dormant liver-stage, drug resistance and disease severity, we compared PvP01 to C127 and the 5 monkey-adapted strains, and generated draft assemblies of patient isolates from Thailand (PvT01) and central China (PvC01). Our sampling focuses on the Asia-Pacific region, where a large burden of *P. vivax* infection lies ^24^. The Indonesian reference provides representation of the island of Papua - the epicentre of multidrug resistance emergence in *P. vivax* ^8^. The draft references from Thailand and Central China provide respective representation of the Mekong region, and the temperate north where long latency phenotypes prevail ^25^.

## Methods

### Samples

Three *P. vivax* field isolates that were judged to be clonal infections following preliminary genomic analysis within the framework of a separate study ^17^ were selected for assembly. The isolates were sourced from a patient presenting at hospital in northern Australia in December 2012 with a recent travel history to Mimika Regency, Papua Indonesia (strain PvP01), and patients presenting with symptomatic infection to local clinics in Nan Province, Thailand in May 2011 (strain PvT01) and Anhui Province, China, in September 2010 (strain PvC01). Patient blood samples were leukodepleted ^26^, and DNA extracted using the QIAamp blood midi kit (Qiagen). All samples were collected with written informed consent from the patients within the framework of previous studies.

### Ethical approval

Ethical approval was provided by the Human Research Ethics Committee of NT Department of Health and Families and Menzies School of Health Research, Darwin, Australia (HREC-09/83), the Mahidol University Faculty of Medical Technology Ethics Committee, Bangkok, Thailand (MUTM 2011-043-03), and the Institutional Review Board of Jiangsu Institute of Parasitic Diseases, Wuxi, China (IRB00004221).

### Sequencing, assembly and annotation

Library preparation and sequencing was performed at the Wellcome Trust Sanger Institute. Genomic DNA was sheared into 300–500 base pair (bp) fragments using ultrasonication (Covaris). Amplification-free Illumina libraries were prepared ^27^ and 75 bp, 100 bp and 250 bp paired end reads were generated on the Illumina GAII, Hi-Seq 2000 v3 and MiSeq platforms respectively, following the manufacturer’s standard cluster generation and sequencing protocols ^28^. Mate-pair libraries with 2–3 kilobase (kb) inserts were additionally prepared for PvP01 and PvT01, using the Illumina mate-pair library preparation kit (v2), and sequenced on the Illumina HiSeq 2500 platform. Prior to assembly, contaminating host–derived sequences were excluded by mapping against the human reference genome (GRCh37: ftp://ftp.1000genomes.ebi.ac.uk/vol1/ftp/technical/reference/) using BWA ^29^ (version 0.7.4). Assemblies were prepared using velvet (version 1.2.07, parameters: -exp_cov auto -ins_length 450 -ins_length_sd 30 -cov_cutoff 8, and using for a kmer of 71) and MaSuRCA ^30, 31^ (version 2.0.3.1, default parameters). Post-assembly genome improvements were undertaken using a range of automated configuration tools including ABACAS (version 2), IMAGE (version 2, iterating k-mers from 71 down 31, 7 iterations), Gapfiller (version 1–11, 14 iteration, parameter n=31) and iCORN (version 2, 7 iterations). PAGIT (version 1) and REAPR (version 1.0.17) were employed to detect assembly errors ^32– 38^. This was followed by visual inspection using ACT ^39^ to identify any further assembly anomalies. Annotation was undertaken initially using the automated algorithms, RATT (version 1) and Augustus (version 2.7, trained on 500 manually curated gene models) ^38, 40, 41^ and further improved by detailed manual inspection performed by an experienced genome curator. PvT01 and PvC01 were annotated using Companion, a new automated annotation tool ^42^. RNA-Seq data from asexual blood stage preparations of 4 *P. vivax* patient isolates from Cambodia (unpublished report, Jessica Hostetler, Lia Chappell, Chanaki Amaratunga, Seila Suon, Thomas D. Otto, Rick Fairhurst and Julian C. Rayner; Accession number ERP017542) was used as supporting evidence to aid the improvement of gene models in PvP01 by manual curation.

For comparative analyses, genome assemblies and gene annotations were sourced for 6 additional *P. vivax* strains; Salvador-I, C127, Brazil-I, India-VII, Mauritania-I and North Korea ^9, 13, 14^. The published version of Salvador-I ^14^ presented in PlasmoDB release 9 was selected for comparison of gene annotations as the additional improvements in release 10 reflected curations performed by the authors. Companion was also used to update the annotation of four previously published genomes (Brazil-I, India-VII, Mauritania-I and North Korea).

### OrthoMCL and *pir* analysis

Comparisons of predicted protein-coding genes between the 9 *P. vivax* assemblies and *P. falciparum* 3D7 (Pf3D7) (geneDB.org) were undertaken using OrthoMCL version 1.4 ^43^ using the default parameter settings. We determined core genes as 1-1 orthologous between *P. vivax* P01 and Pf3D7, in total 4465.

Cluster analysis based on structural and sequence homology was undertaken to compare the subfamily organization of the *pirs* in the partial (Salvador-I) versus more complete (PvP01) reference. All PIR encoded protein sequences in Salvador-I and PvP01 with length greater than 150 amino acids and not flagged as pseudogenes were included in the analysis. Low complexity regions were excluded using the SEG program ^44^. The relatedness between sequences was assessed using BLASTp (parameters -F F -e 1e-6), and the results were visualized as a network constructed in Gephi ^45^. After provisional assessment of cluster resolution at different thresholds, a cut-off of 25% of the global similarity was selected for distinguishing different clusters (subfamilies). To aid comparison against the new PIRs identified in PvP01, the Salvador-I PIRs were colour-coded according to the subfamily classification proposed by Lopez *et al* ^23^.

Further investigation of the diversity and relatedness amongst the PIRs was undertaken using the PIR sets from PvP01, PvT01, PvC01, Salvador-I and Brazil-I. Exclusion of proteins with less than 150 amino acids, filtering of low complexity sequences and relatedness analysis using BLASTp were performed as described above. A network was constructed from the BLAST output using tribeMCL with an inflation of 1.5 ^46^. To aid visualization, clusters with less than 15 PIRs were excluded.

## Dataset validation

The PvP01 assembly was generated as a new reference sequence and is thus a higher quality, more accurately annotated assembly than PvC01 and PvT01, which were both created as draft assemblies for comparative purposes. The PvP01 assembly quality is greatly improved over the previous Salvador-I reference genome, with fragmentation reduced to <250 scaffolds amongst other features ( Table 1). At 29 megabases (Mb), the assembly is notably larger than Salvador-I (27 Mb), mainly due to newly assembled subtelomeric sequences. A complete mitochondrial sequence (5 kb) and partial apicoplast sequence (29.6 kb) are also available. As in *P. falciparum* ^47^, the apicoplast reference will facilitate efforts to identify geographic surveillance markers for *P. vivax*.

**Table 1.** Features of the new *P. vivax* assemblies against Salvador-I.

Genome features	PvP01 ^a^	PvC01	PvT01	Salvador-I ^b^
**Nuclear genome**				
Assembly size (Mb)	29.0	30.2	28.9	26.8
Coverage (fold)	212	56	89	10
G + C content (%)	39.8	39.2	39.7	42.3
No. scaffolds assigned to chrom.	14	14	14	30
No. unassigned scaffolds	226	529	359	2745
No. genes ^c^	6,642	6,690	6,464	5,433
No. *pir* genes	1,212	1,061	867	346
**Mitochondrial genome** ^**d**^				
Assembly size (bp)	5,989	-	-	5,990
G + C content (%)	30.5	-	-	30.5
**Apicoplast genome**				
Assembly size (kb)	29.6	27.6 ^e^	6.6 ^f^	5.1 ^g^
G + C content (%)	13.3	12.7	19.7	17.1
No. genes	30	3	0	0

^a^ Genome version 1.09.2016 ^b^ Published reference sequence ^14^ ^c^ Including pseudogenes and partial genes, excluding non-coding RNA genes. ^d^ Mitochdondrial genome is not present in PvT01 and PvC01 ^e^ scaffold PvC01_00_191 ^f^ scaffold PvT01_00_162 ^g^ Partial apicoplast sequence of Salavador-I reference assembly has been published (scaffolds AAKM01000417, AAKM01000371)

**Figure 1.**
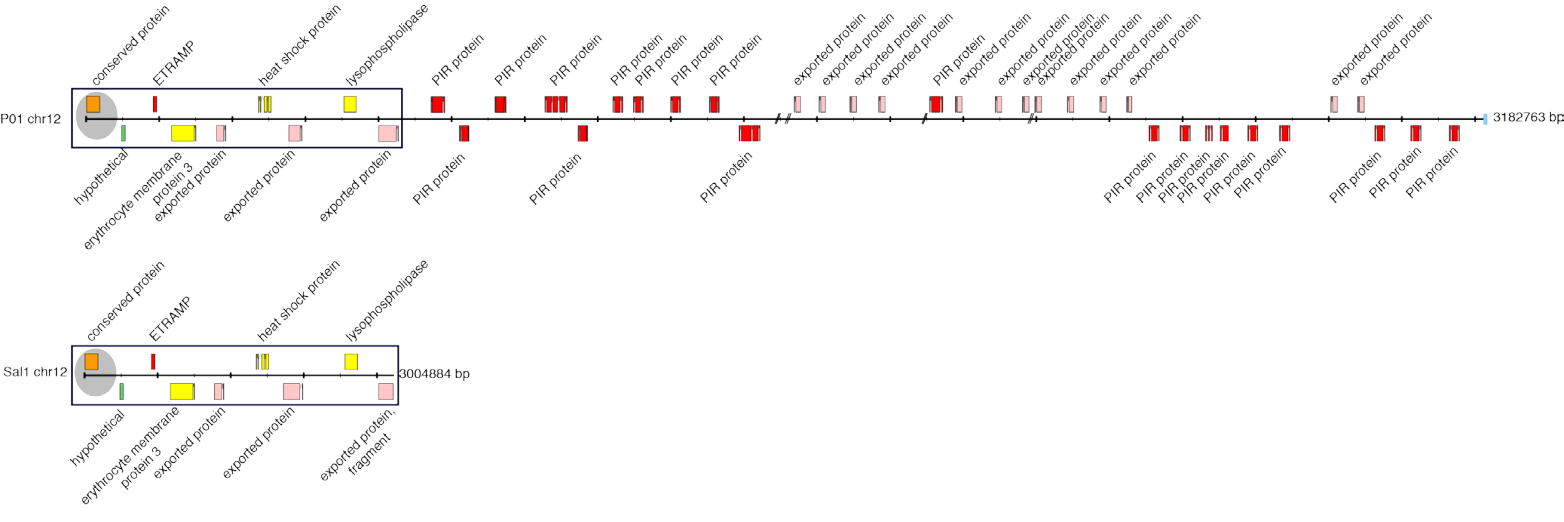
Organization of the subtelomeric regions of chromosome 12 of the PvP01 and Salvador-I *P. vivax* references illustrating the higher assembly quality of PvP01. The order and orientation of the genes in the 3’ subtelomeric region of chromosomes 12 of PvP01 (top) and Salvador-I (bottom) are shown. Exons are shown in coloured boxes, with introns illustrated by linking lines. Gaps in PvP01 are indicated with a forward slash (“/”). The blue box indicates the start of the telomeric heptamer repeats. The shaded (grey) areas mark the start of the conserved core of the chromosome that shares synteny with other *Plasmodium* species (e.g. *P. falciparum*). The black box shows the syntenic area of PvP01 and Salvador-I. The last gene in this syntenic area is fragmented in Salvador-I.

Whilst the assembly quality in the core region is high in Salvador-I ^14^, PvP01 displays improved gene models and has more complete subtelomeres. Figure 1 provides a schematic of the right-hand end of chromosome 12 from PvP01 and Salvador-I, illustrating the generally greater extension into the subtelomeric regions of chromosomes in PvP01. Furthermore, owing to detailed manual curation and continuous maintenance within the GeneDB framework, the level of gene annotation in the core genome of PvP01 greatly exceeds that of the other available *P. vivax* assemblies. The asexual stage *P. vivax* RNA-Seq data enabled correction of the structure of 377 genes. Of the 4577 core *P. vivax* genes with 1:1 orthologues in *P. falciparum*, 3318 genes were transcribed with RPKM (reads per kilobase of transcript per million mapped reads) values greater than 15, and contained a total of 4887 splice sites. Of these splice sites, a total of 4845 (99.1%) were confirmed by ≥ 10 reads, highlighting the high quality of the structural annotation. Whereas the published Salvador-I reference includes functions attributed to a total of 1783 (38.0%) core genes ^14^, we have been able to expand this to 2848 (58.6%) in PvP01, as of the latest GeneDB release (1st September 2016). Ongoing curation on PvP01 will yield further improvements to the annotation statistics, and progress is highlighted in Table 2, which summarizes annotation changes over a 12 month period between GeneDB releases in 2015 and 2016. To date, a total of 1209 genes have been identified in PvP01 that were either completely absent from Salvador-I or have arisen by splitting gene structures that were falsely joined previously ( Table 1). Although the majority of newly identified genes belong to subtelomeric gene families, we confirmed the recently identified EBP2 (erythrocyte binding protein 2, PVP01_0102300) and RBP2e (reticulocyte binding protein 2e, PVP01_0700500) genes ^11^. These genes are members of families encoding proteins implicated in host cell recognition during red blood cell (RBC) invasion, and present potential vaccine targets ^48– 51^.

**Table 2.** Annotation changes in *P. vivax* P01 from 1 ^st^ of September 2015 until 27 ^th^ of September 2016.

Annotation event type	PvP01 ^a^
Assigned or updated product	408
Product updated from “conserved Plasmodium protein, unknown function”	107
Updated GO term	597
Linked to publication	291
All unique genes with new functional annotations, e.g. EC number, gene name	608
All unique genes with new structural annotations	50

^a^ Genome version 1.09.2016

**Figure 2.**
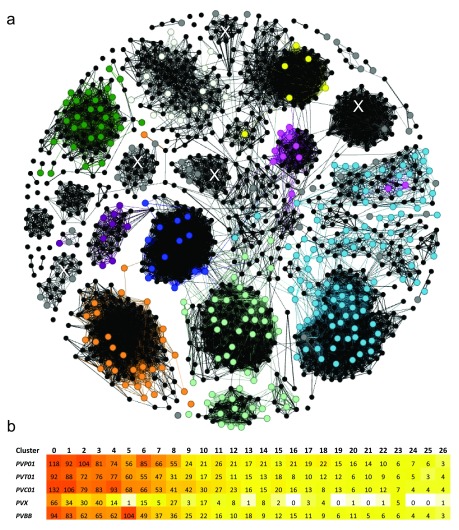
Cluster analysis illustrating the relatedness between the PIR proteins in PvP01 versus Salvador-I (a), and the stability of the major clusters in several other *P. vivax* assemblies (b). Panel a) presents a network illustrating the relatedness between the 1063 PIR proteins of PvP01 and 341 PIRs of Salvador-I (Sal-I) with length greater than 150 amino acids. The PvP01 PIRs are illustrated by black dots (nodes). The Sal-I PIRs are illustrated by coloured dots with colour-coding according to the subfamily classification of Lopez *et al* ^23^ as follows; purple = A, pink = B, pale green = C, red = D, pale blue = E, orange = G, green = H, blue = I, white = J, yellow = K , and grey = unassigned genes. Two nodes (PIRs) are connected if they have a global similarity of at least 25%. With the exception of a few proteins, the majority of Sal-I PIRs demonstrate clustering consistent with the classification of Lopez *et al*. Five new, interconnected clusters comprising previously unassigned Sal-I PIRs are denoted with a white “X”. In Panel b, a heat map summarises the number of PIRs assigned to the 27 major clusters (minimum 15 PIRs in total) in five geographically divergent *P. vivax* strains; PvP01 (Papua Indonesia), PvT01 (Thailand), PvC01 (Central China), Sal-I (El Salvador) and Brazil-I (Brazil). With the exception of Sal-I, which displayed fewer genes than the other isolates in several of the major clusters, the isolates demonstrated similar numbers of genes in most clusters.

As summarised in Table 3, the comparatively high assembly quality in the subtelomeres of PvP01 greatly expanded the repertoire of genes belonging to multigene families in these chromosome regions. Notably, more than 1200 *pir* genes were identified in PvP01 versus 346 in Salvador-I. To generate a snapshot of the diversity and structural organization of this expanded gene family in *P. vivax*, we conducted cluster analysis of the PIRs in PvP01 with comparison to previous homology classifications performed by Lopez *et al* on the partial set of PIRs from Salvador-I ^23^. As illustrated in the network diagram in Figure 2a, the main subfamily clusters defined in earlier classifications are expanded but, on addition of the new PvP01 PIRs, the clusters remained moderately stable with no pooling between or sub-structure within subfamilies. However, the new PvP01 PIRs reveal several large subfamilies containing just 1–4 Salvador-I genes that were previously unclassified ( Figure 2a). Additional investigation with the PvC01, PvT01 and Brazil-I assemblies using tribeMCL (also used in Lopez *et al)* confirmed the stability of the new subfamilies identified in PvP01 across a geographically divergent collection of isolates ( Figure 2b). The analysis conducted here provides a broad overview of the diversity and relatedness amongst the expanded *P. vivax pir* gene sets, however further investigation beyond the scope of this study will be required to provide detailed characterisation of this family and its contribution to virulence and pathophysiology.

**Table 3.** Number of most abundant genes in the subtelomeres in the genomes of Salvador-I, PvP01, PvT01 and PvC01.

	Description	Sal-I ^a^	PvP01 ^b^	PvC01	PvT01
**Multigene family**	PIR protein ^c^	346	1212	1061	867
	tryptophan-rich protein ^d^	34	40	40	40
	lysophospholipase ^e^	11	10	9	8
	STP1 protein ^f^	9	10	11	3
	early transcribed membrane protein (ETRAMP)	10	9	9	9
	Plasmodium exported protein (PHIST), unknown function ^g^	64	84	22	23
	reticulocyte binding protein (RBP)	9 ^h^	9 ^h^	9	8
**Other genes**	Plasmodium exported proteins of unknown function ^i^	23	447	266	261
**Total**	n/a	497	1812	1427	1219

Numbers include pseudogenes and partial genes ^a^ Published reference sequence ^14^ ^b^ Genome version 1.09.2016 ^c^ Other names include VIR protein and Pv-fam-c protein ^d^ Other names include Pv-fam-a, trag and tryptophan-rich antigen ^e^ Other names include PST-A protein ^f^ Other names include PvSTP1 ^g^ Other names include Phist protein (Pf-fam-b) and RAD protein (Pv-fam-e) ^h^ Includes RBP2e (PVP01_0700500) that was not present in the Salvador-I assembly. RBP1b (PVP01_0701100) is complete in PvP01. In Salvador-I RBP1b consists of two partial genes (PVX_098582, PVX_125738) ^i^ Other names include Pv-fam-d protein and Pv-fam-c protein

The PvP01 reference is an important new resource for the vivax research community. It will support studies of the complex subtelomeric regions and provide insights into the mechanisms by which the gene families in this region contribute to virulence-associated functions. It will also allow investigation of an array of other biological functions that will expand with continual improvements in annotation in the core genome. PvP01, PvC01 and PvT01 add new geographic locations to the collection of *P. vivax* assemblies, facilitating biological studies of the diversity of this phenotypically divergent species.

## Data availability

The raw sequence data for PvP01, PvT01 and PvC01 can be retrieved from the European Nucleotide Archive; sample accession numbers PvP01 ERS017708, ERS312161 3kb ERS328510, PvT01 ERS055881, ERS312160 3kb ERS328509 and PvC01 ERS407449. The assemblies can be found under the study PRJEB14589. The individual accession numbers are PvP01 (chromosomes: currently in submission to EBI, files on ftp, contigs: FLZR01000001-FLZR01000226), PvT01 (chromosomes LT615239-LT615252, contigs: FLYH01000001-FLYH01000360) and PvC01 (chromsomes LT615256-LT615269, contigs: FLYI01000001-FLYI01000530). PvP01 is maintained in GeneDB: http://www.genedb.org/Homepage/PvivaxP01 and updates are synchronized to PlasmoDB.

This section will be updated with accession numbers for PvP01 chromosomes onces available.
