# Supplementary material for: A new Plasmodium vivax reference sequence with improved assembly of the subtelomeres reveals an abundance of pir genes
Source: Wellcome Open Res. 2016 Nov 15;1:4. [Version 1] doi: 10.12688/wellcomeopenres.9876.1 (PMC5172418; doi:10.12688/wellcomeopenres.9876.1)
Supplement: Peer review history

# Peer review: Referee response for version 1

**DOI:** 10.21956/wellcomeopenres.10647.r18200

**Published:** 2016-12-19

**Authors:** Liwang Cui

**Affiliations:** 1 Department of Entomology, Pennsylvania State University, State College, PA, USA

This work describes *de novo* assemblies of three new *P. vivax* genomes and comparison with the reference Sal I genome and other Pv genomes. Compared with previous annotation of the reference genome, the new assembly of the PvP01 genome for an isolate from Papua Indonesia has reduced the total scaffolds from over 2500 in SalI to 226 (+14). Major improvements are in the subtelomeric regions, where a significantly increased number of pir genes have been discovered. This more in-depth study of the Pv genome and manual curation of genes provide a better resource for biological studies of the vivax parasite.

 Comments:

1. Abstract: The quality of the PvP01 assembly is improved greatly over Salvador-I, with fragmentation reduced to 226 scaffolds.  Perhaps “with fragmentation reduced to 226 unassigned scaffolds in addition to the 14 chromosomal scaffolds” will be more accurate?
2. Does the “results” section begin at “Dataset validation”?
3. Table 1 presented comparison of the genomes the three new sequences with that of Sal I. The PvC01 and PvT01 sequences contained more assigned scaffolds – are these located mostly in the telomeric regions?
4. A more detailed comparison of the temperate strain PvC01 with the tropical strains would be more useful.  A big-picture type perspective on the C01 and T01 would be nice.
5. Figure 1 illustrates the extension of the assembled sequences in the subtelomeric region of chrom12 as compared to that in SalI. Are the gap junctions verified by PCR? Also, the PvP01 also has quite some gaps – how are these assembled and verified?
6. The network presentation of the Pir genes is interesting – A link to the alignment of the sequences or a phylogenetic tree-type of presentation (as supplements) would be very useful.

I have read this submission. I believe that I have an appropriate level of expertise to confirm that it is of an acceptable scientific standard.

# Peer review: Referee response for version 1

**DOI:** 10.21956/wellcomeopenres.10647.r18268

**Published:** 2016-12-12

**Authors:** Richárd Bártfai

**Affiliations:** 1 Department of Molecular Biology, Radboud University Nijmegen, Nijmegen, Netherlands

In this manuscript, Auburn and colleagues describes the generation and initial analysis of a new *P. vivax* reference sequence. The authors extensively sequenced a single-clone field isolate from Papua Indonesia using Illumina technology. Assembly of these sequence reads lead to a much less fragmented and better covered core genome sequence. Furthermore, the improved assembly and annotation resulted in a much more complete overview on the subtelomeric multi gene families in this and two other isolates from China and Thailand.

 Reference genomes are the foundation for all genomic, transcriptomic and proteomic studies. Therefore, this new reference genome sequence is very welcome and will undoubtedly fuel the exploration of the biology and pathogenesis of *P. vivax*. While it is always difficult to access the quality of such assemblies based on description only it is conceivable that the 20x increase in coverage and the use of various post-assembly improvement tools have resulted in considerably better genome sequence. Furthermore, the manual curated gene models and functional classifications bring substantial added value to this work.

 Overall this study is well executed and the manuscript is well-written. I have only some minor suggestions for improvement:

- It would be important to clarify in the manuscript why PvP01 has been chosen to be the new reference “strain”.
- Sequencing and annotation of multigene families is challenging. To fully exclude the possibility that the 5 new clusters of PIR proteins identified in this study are the result of incorrect sequence assembly it would be relevant to PCR amplify and sequence a representative member from each of these families.
- In the abstract the authors state that the new reference genome contains 226 scaffolds, while according to table 1 it appears to be 226+14. Please double-check.
- I do not find Table 2 particularly useful/informative. It is basically a tribute to a huge amount of work.
- It might not be formally required to include a subheading “Results” in Welcome Open Research data notes, but nonetheless it would be nice to know where the description of the results begins.
- It would bring added value to this article if Table 3 would be extended by description of all and not only the subtelomeric gene families (Table 2 in Tachibana *et al*., 2012 ^1^ could provide a nice example). Instead of the extensive footnotes an extra column could be included for alternative names.
- In Table 3 it is unclear if other genes includes only “Plasmodium exported protein of unknown function” or also other proteins. If there are indeed couple of hundred of these proteins encoded in the PvP01 genome and they localize to the subtelomeric regions as Figure 1 suggests, it would be perhaps relevant to discuss them as a gene family. It could even be worthwhile to perform a cluster analysis on this “gene family” similar to the one performed on PIR proteins.
- On Figure 2B it would be useful to indicate the correspondence between the cluster numbers of this study and the former classification (A-K). Similarly it would be informative to indicate the cluster numbers on Figure 2A.
- From Figure 2B it seems that cluster 5 PIR gene subfamily has expanded (substantially more numerous) in the Brazilian isolate. Something perhaps worthwhile mentioning/discussing.

I have read this submission. I believe that I have an appropriate level of expertise to confirm that it is of an acceptable scientific standard.

## References

[1] TachibanaSSullivanSAKawaiSNakamuraSKimHRGotoNArisueNPalacpacNMHonmaHYagiMTouganTKatakaiYKanekoOMitaTKitaKYasutomiYSuttonPLShakhbatyanRHoriiTYasunagaTBarnwellJWEscalanteAACarltonJMTanabeK: Plasmodium cynomolgi genome sequences provide insight into Plasmodium vivax and the monkey malaria clade. *Nat Genet*.2012;44(9) : 10.1038/ng.2375 1051-5 10.1038/ng.2375 22863735PMC3759362
